# N6-isopentenyladenosine induces cell death through necroptosis in human glioblastoma cells

**DOI:** 10.1038/s41420-022-00974-x

**Published:** 2022-04-07

**Authors:** Cristina Pagano, Giovanna Navarra, Laura Coppola, Giorgio Avilia, Olga Pastorino, Rosa Della Monica, Michela Buonaiuto, Giovanni Torelli, Pasquale Caiazzo, Maurizio Bifulco, Chiara Laezza

**Affiliations:** 1 Department of Molecular Medicine and Medical Biotechnology, University of Naples “Federico II,”, Naples, Italy; 2 CEINGE—Biotecnologie Avanzate, Via Gaetano Salvatore, 486, 80145 Naples, Italy; 3 Neurosurgery Unit A.O. San Giovanni di Dio e Ruggi d’ Aragona—Salerno’s School of Medicine Largo Città di Ippocrate, 84131 Salerno, Italy; 4 Neurosurgery, Unit A.O. “A.Cardarelli”, Naples, Italy; 5 Institute of Endocrinology and Experimental Oncology (IEOS), National Research Council (CNR), Naples, Italy

**Keywords:** Cancer therapeutic resistance, CNS cancer

## Abstract

Targeting necroptosis is considered a promising therapeutic strategy in cancer, including Glioblastoma Multiforme (GBM), one of the most lethal brain tumors. Necroptosis is a mechanism of programmed cell death overcoming the apoptosis resistance mechanism underlying GBM tumorigenesis and malignant progression. N6-isopentenyladenosine (iPA), adenosine modified with isoprenoid derivative, displays antitumor activity in different cancer models. In previous studies, we demonstrated that iPA interferes with EGFR signaling reducing glioma cell viability. Here, we show that iPA induces necroptosis in glioblastoma cell lines and in primary cells established from tumor explants, without affecting the viability of non-cancerous brain cell lines, (Normal Human Astrocyte). The activation of RIP1, RIP3, and MLKL and the upregulation of necrosome formation were increased upon iPA treatment while caspase-3, caspase-8, and PARP were not activated in GBM cells. Co-treatment with specific necroptosis inhibitor necrostatin-1 (Nec-1) or Necrosulfonamide (NSA) prevented cell death caused by iPA treatment while the general caspase inhibitor Z-VAD-fluoromethylketone (z-VAD-fmk) did not elicit any effect, suggesting that this molecule induces caspase-independent necroptosis. These results suggest that iPA treatment can be able to bypass the apoptosis resistance mechanism in glioblastoma thereby offering higher therapeutic efficacy.

## Introduction

Glioblastoma Multiforme (GBM) is the deadliest form of cancer of the human central nervous system, characterized by a high proliferation rate, invasion into surrounding normal brain tissues with a median survival for patients of 12–15 months and a 5-year survival rate of <5% [1]. The high frequency of molecular alterations (p53, RB, unmethylated MGMT) renders GBM poorly susceptible to cytotoxic therapies and highly resistant to standard chemotherapy and radiotherapy [2]. Recent data have described that some drugs may represent promising chemotherapy for the GBM treatment, able to inhibit the growth of cancer cells by triggering cell death mechanisms, interfering with the transcriptional and epigenetic pathway, or inhibiting the self-renewal of GBM stem cells. Furthermore, the biology of GBM is not yet fully known, for this reason, it is important to identify new targets as well as molecular mechanisms that can suggest alternative therapeutic strategies. A further obstacle to effective therapy is the GBM inter- and intratumoral heterogeneity [3]. GBM is made up of several cell types characterized by the ability to promote cancer progression in various ways [4]. Glioma cancer cells have different molecular characteristics, among which the amplification of the epidermal growth factor receptor (EGFR) gene is frequently found. In addition, EGFR can be characterized by the modification of some of its domains, such as the truncation of the extracellular domain that gives rise to the mutated form EGFRvIII. Both alterations promote tumor growth and endurance through the overactivation of downstream pro-oncogenic signalling pathways [5]. Although, both receptors have been linked to GBM resistance to chemotherapy and to apoptosis mechanism. Indeed, a key role in both gliomagenesis and the aggressive characteristics of GBM is the deep dysregulation of apoptosis signaling [6], which is the most known form of programmed cell death, often targeted in cancer therapy approaches [7]. Emerging evidence suggests that GBM cells evade elimination when treated with both conventional and targeted therapies by overriding apoptosis [8]. Therefore, the development of new approaches targeting compensatory/resistance mechanisms may be required to achieve a more robust antitumor effect. Necroptosis is a form of regulated cell death process induced when apoptosis fails. It is characterized by the disruption of the plasma membrane and the key molecules required to execute this process are serine–threonine kinases RIP1 and RIP3. Activation of these kinases determines the formation of necrosome and the recruitment of MLKL, which is then phosphorylated. Activated MLKL translocates to the plasma membrane causing its rupture and then necrotic cell death [9]. N6-isopentenyladenosine (iPA) is a cytokinin regulating plant cell growth and differentiation. In addition, iPA is present in mammalian cells bound to tRNA. iPA has shown antiproliferative and proapoptotic effects against a broad variety of tumors in vitro and in vivo [10, 11]. This compound is able to inhibit farnesyl pyrophosphate (FPPS) and protein prenylation [12]. More recently, it has been shown that iPA inhibits in vitro and in a mouse model vivo the angiogenic processes [13] and in vitro the vasculogenic mimicry [14]. In the present study, we observed that iPA induced necroptosis in GBM cells lines and primary GBM cells derived from tumor biopsies of patients affected. iPA treatment caused the increase of key markers activation of necroptosis after 18 h, as RIP1, RIP3, and MLKL form necrosome in the cytosol. We highlighted the HMGB1 release and the augmented expression levels of PUMA which has been recently described to be involved in the necroptotic process [15]. Inhibition of the kinase activity of RIP1 by necrostatin-1 (Nec-1) and MLKL activity by Necrosulfonamide (NSA) blocks necroptosis induced by iPA.

## Results

### iPA reduces cell viability in a dose- and time-dependent manner for GBM cells

**Fig. 1:**
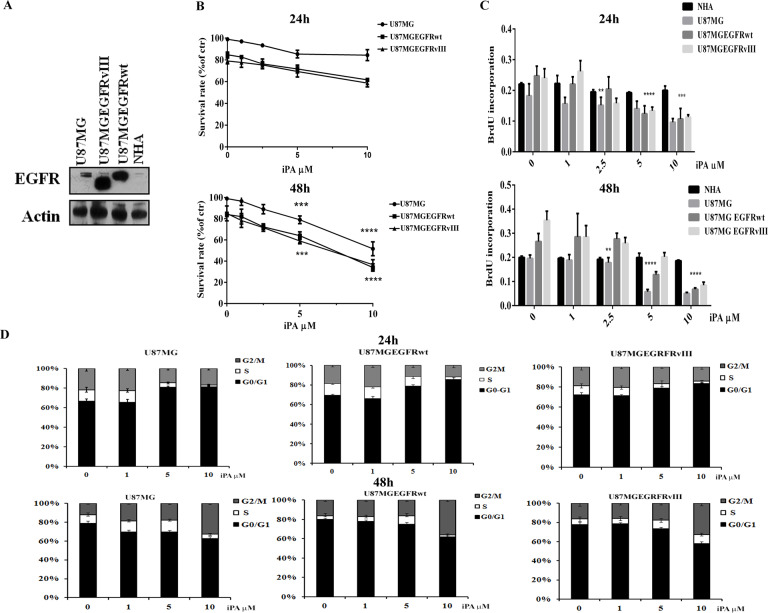
Antiproliferative effect of iPA in GBM cells. **A** Western Blot assay showing the different expression levels of EGFR in U87MG, U87 EGFRvIII, U87 EGFRwt, and NHA cell lines. **B** Graphic representation of results from MTT assays to determine cell viability of U87MG, U87EGFRvIII, and U87EGFRwt treated with different concentrations (0–10 µM) of iPA for 24 h and 48 h. **C** Antiproliferative effect of iPA on U87MG, U87 EGFRvIII, U87 EGFRwt GBM cell lines, and NHA cells as detected by BrdU assay after 24 h and 48 h of treatment. **D** Graphic representation of cell cycle distribution obtained using PI staining and flow cytometry. Data points are the percentage of cells in G0/1, S, and G2/M in U87MG at 24 h and 48 h after iPA treatment. The data are presented as mean ± SD of at least three independent experiments. **p < 0.01, ***p < 0.001, ****p < 0.0001 versus control, represented by cells treated with vehicle (DMSO).

GBM is characterized by different molecular characteristics among which the amplification of the epidermal growth factor receptor (EGFR) in association with a variant of receptor EGFRvIII may cause drug and apoptosis resistance mechanisms hindering therapeutic success. To represent this molecular characteristic we used glioblastoma cell lines, U87MG, and the same ones engineered to overexpress wild-type (wt) EGFR or EGFRvIII (Fig. 1A) to explore the iPA antiproliferative effect. We carried out an MTT and a BrdU incorporation assays at increasing concentrations of iPA (0–10 µM) at 24 h and 48 h. As shown in Fig. 1B, C, iPA induced inhibition of the cell viability and DNA synthesis in a dose and time-dependent manner compared to cells treated with vehicle DMSO, showing a IC50 of 3 µM for U87MG, 4.3 µM for U87EGFRwt and 7.1 µM for U87EGFRvIII after 48 h of treatment. No significant cytotoxic effects were found at 1 and 2.5 µM, while at 24 h we observed a poor inhibition of cell proliferation at 10 µM in U87MG cells and over-expressing EGFRwt/EGFRvIII cells (Fig. 1B). Moreover, iPA did not elicit a significant reduction in the cell proliferation of the counterpart normal GBM cells, the normal human astrocytes (NHA) cells (Fig. 1C). Reduced GBM cell growth after iPA treatment was also due to a perturbation of cell cycle progression (Fig. 1D). The cell cycle analysis of the GBM cell lines revealed that exposure to iPA a 10 µM for 24 h caused an increase in G_0_/G_1_ phase while at 48 h a reduction of GBM cells in G_0_–G_1_ phase and a significant increase of cells in the G_2_/M phase as compared to untreated cells (Fig. 1D).

### iPA induces necroptosis in GBM cells

**Fig. 2:**
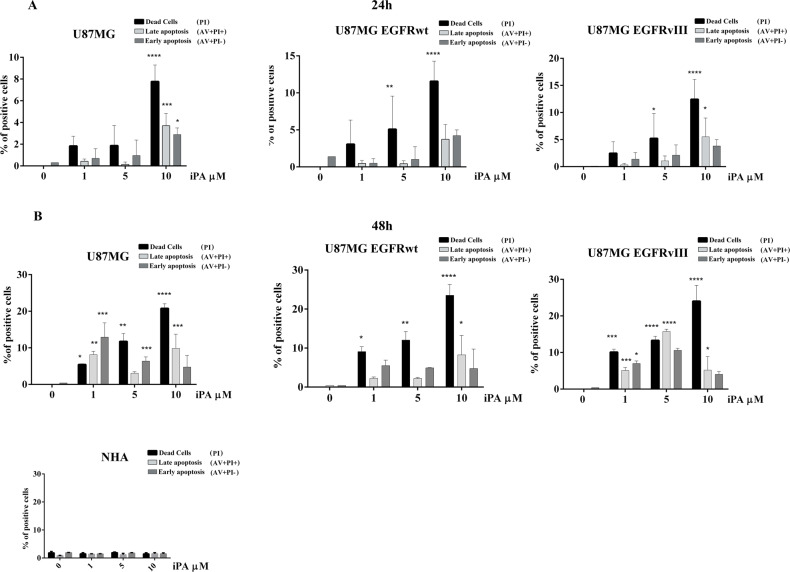
iPA induces cell death. GBM stabilized cell lines and normal control NHA cells were treated with 1, 5, and 10 µM of iPA for 24 h (**A**) and 48 h (**B**). Cell death was determined by flow cytometry followed by Annexin V/PI staining. Early Apoptosis: Annexin V-positive cells (AV+/PI−); Necrosis: PI-positive and Annexin V-negative cells (AV−/PI+); and Late apoptosis (AV+/PI+). The data are presented as mean ± SD of at least three independent experiments. *p < 0.05, **p < 0.01, ***p < 0.001, ****p < 0.0001 versus control, represented by cells treated with vehicle (DMSO).

To investigate if, in addition to the inhibition of DNA synthesis, iPA is also able of inducing cell death, we performed analysis by Annexin-V and PI double staining of the GBM cell lines at 24 h and 48 h of treatment by flow cytometer (Fig. 2A, B). We observed a dose-dependent induction of necrosis (AV−/PI+(Necrosis): 21.7% ± 2.27 for U87MG; 21.8% ± 4.5 for U87EGFRwt; 18.1% ± 5.2 U87EGFRvIII) and late apoptotic (AV+/PI+(Late Apoptosis): 7.3% ± 4.2 for U87MG; 4.5% ± 5.02 for U87EGFRwt; 4.7% ± 4.0 for U87EGFRvIII) rates at 10 µM of iPA after 48 h of treatment (Fig. 2B); the necrosis rate was prevalent compared to the late apoptosis rate (Fig. 2B). Interestingly apoptotic events were barely detectable in NHA cells (Fig. 2B). These results suggest induction of necroptosis by iPA at 10 μM.

**Fig. 3:**
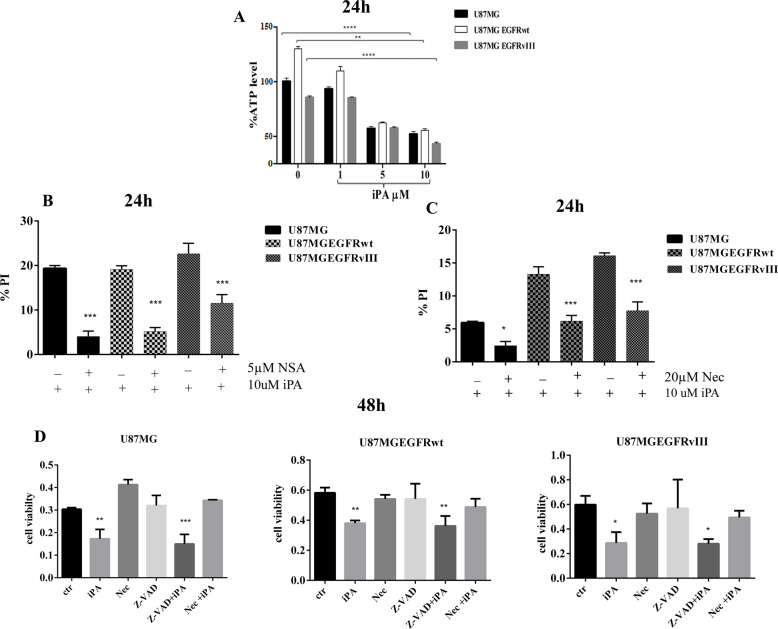
iPA induces necroptosis. **A** Plot showing the reduced ATP levels after 24 h of treatment with iPA 10 µM in U87 GBM stabilized cell lines. **B**, **C** Plot showing the % of PI-positive cells. Stabilized GBM cell lines were pretreated for 1 h with Necrosulfonamide (NSA) (5 μM) (**B**) and Necrostatin-1 (Nec-1) (20 μM) (**C**) and then analyzed through PI staining after 24 h of iPA treatment (10 μM). **D** Plot showing the % of cell viability in U87 GBM cell lines after 48 h of treatment with iPA (10 μM), RIP1 inhibitor Nec-1 (20 μM), Caspase inhibitor z-VAD (10 μM), and two combinations of these drugs: z-VAD (10 μM) + iPA (10 μM) and Nec-1 (20 μM) + iPA (10 μM). Specifically, GBM cells were pretreated with z-VAD or Nec-1 for 1 h, followed by treatment with iPA 10 μM for 48 h. The data are presented as mean ± SD of at least three independent experiments. *p < 0.05, **p < 0.01, ***p < 0.001, ****p < 0.0001 versus control, represented by cells treated with vehicle (DMSO).

To further investigate the pathway involved, we performed molecular characterization experiments, including: (a) depletion of intracellular ATP levels; (b) PI staining for necrotic cells and inhibition by necroptosis inhibitors, NSA and Nec-1; (c) activation of necroptosis markers; and (d) necrosome formation. Regarding the first point, a significant decrease in intracellular ATP levels was observed in the iPA-treated cells at 24 h compared to untreated cells in a dose-dependent manner (Fig. 3A). Because we observed a significant and sturdy effect of iPA at 10 µM, we performed the next experiments at this concentration. In opposition to apoptosis, necroptosis is characterized by plasma membrane disintegration leading to cell swelling and loss of nuclear organization. For this reason, we verified the PI uptake which indicates a loss of plasma membrane integrity and can thus be used to distinguish necroptotic cells from apoptotic cells. After 24 h of iPA treatment, the PI staining rate was 24.8% ± 4.4 for U87MG, 20.6 ± 1.3 for U87EGFRwt and 21.1 ± 0.6 for U87EGRvIII (Fig. 3B, C). The PI uptake was inhibited at 11.7% ± 0.1, 7.75 ± 2.7, and 13.9 ± 1.4 rates respectively after the GBM cell lines were pretreated 1 h with an inhibitor of necroptosis marker MLKL, NSA at 5 µM (Fig. 3B). We also observed an inhibition in the PI uptake in the GBM cell lines pretreated for 1 h with RIP1-dependent programmed necrosis, (Nec-1) at a concentration of 20 µM (Fig. 3C). To further explore the mechanism underlying iPA-induced GBM cell death, apoptosis and necroptosis inhibitor were applied to cell cultures 1 h before iPA treatment. As shown in Fig. 3D, after treatment with iPA for 48 h in the absence of inhibitors, MTT assays indicated that the cell viability decreased significantly. Relative to this effect, the survival rate was increased after pretreatment with the Nec-1. In contrast, iPA-induced cell death was not attenuated by pretreatment with z-VAD-fmk, a broad-spectrum inhibitor of caspase-dependent apoptosis. These results indicate that under the above experimental conditions, iPA-induced cell death through necroptosis.

### The necroptosis induction occurs by RIP1/RIP3/ MLKL pathway

**Fig. 4:**
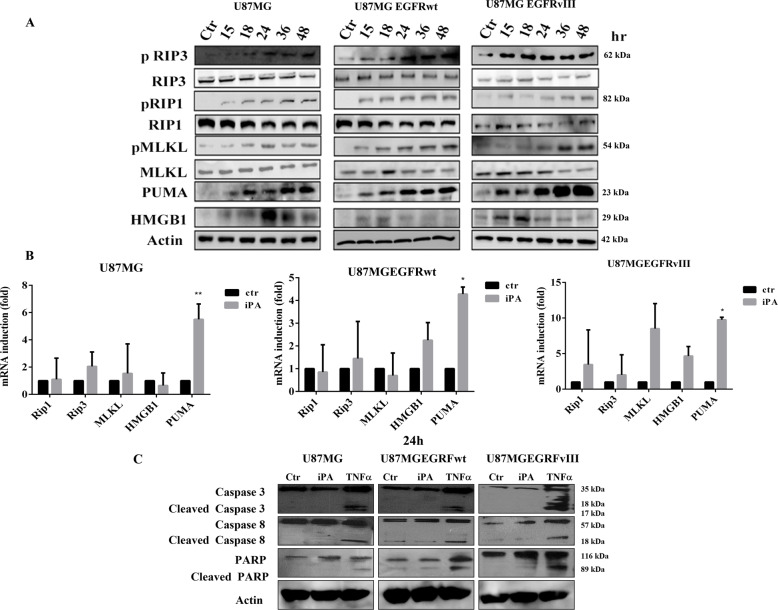
iPA induces phosphorylation of necroptosis’s markers. **A** Representative Immunoblotting images of Necroptosis protein levels. After treatment with iPA 10 µM at different timepoints (15–48 h), the activation levels of pRIP1, pRIP3, and p-MLKL and the protein levels of PUMA, HMGB1 were increased. **B** Relative expression levels of Necroptosis markers after iPA treatment. Each bar represents the SD value from three independent replicates. **C** Representative Immunoblotting images of Caspase-3, Caspase-8, and PARP1 protein levels after treatment with iPA 10 µM at 24 h. The data are presented as mean ± SD of at least three independent experiments. *p < 0.05, **p < 0.01, ***p < 0.001, ****p < 0.0001 versus control, represented by cells treated with vehicle (DMSO).

**Fig. 5:**
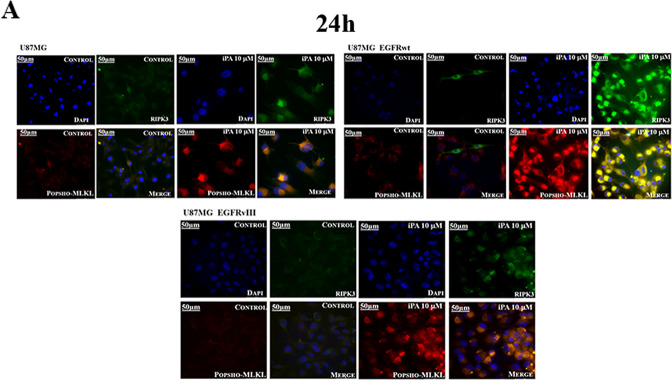
iPA increases formation of necrosomes. **A** Immunofluorescence staining of U87 GBM cell lines with RIPK3 (green) and p-MLKL (red) after treatment with iPA 10 µM at 24 h. The nuclei were stained blue with Hoechst. The merged images show the necrosome formation in yellow due to the signal overlap of RIPK3 and p-MLKL (green + red). The data are presented as mean ± SD of at least three independent experiments.

RIPK1/RIPK3/MLKL activation is an important step in necroptotic signaling pathways, therefore, we analyzed both the expression and activation of RIP1, RIP3, and MLKL, components of necrosome complex [9], by means of western blotting and qRT-PCR assays. We ascertained an increase in the phosphorylation status of necroptosis markers after 15 h of iPA treatment (Fig. 4A and Sup.1), with the use of specific antibodies in GBM cell lines treated with iPA at 10 µM for 15 h compared to untreated cells, a result that increases at 48 h, when we also revealed the highest percentage of necrotic cells (Fig. 2B). The activation of these markers occurs together with the increase of HMGB1 protein at 24 h for U87MG and at 18 h for the cells overexpressing EGFR/EGFRvIII and PUMA induction which, recently, has been demonstrated to contribute to the execution of necroptosis (Fig. 4A and Sup.1) [12]. Consistent with this, we found through qRT-PCR assay a significant increase of expression of PUMA in cells treated for 24 h with iPA 10 µM compared to untreated cells (Fig. 4B) while we did not find variability in the expression levels of the markers. To further explore the cell death mechanism, we investigated caspase-3, -8, and PARP activation by immunoblotting assay. During necroptosis, activation of caspase-8 was reported to be inhibited [16]. In the present experiment, the amount of the cleaved caspase-8 was not changed in treatment with iPA compared to the untreated group (Fig. 4C). Moreover, the levels of the apoptosis-related proteins including cleaved caspase-3, and PARP were also inhibited in cells treated with iPA (Fig. 4C). On the other hand, GBM cells treated with TNF-α an inducer of apoptosis, activate caspases-3, 8, and PARP1. These data indicated that the main mechanism for iPA in causing cell death of GBM cells is caspases-independent necroptosis. Finally, through immunofluorescence analysis, we revealed the colocalization of pMLKL and RIP3 in GBM cells treated with iPA for 24 h as compared to control cells. As depicted in Fig. 5A, in iPA-treated GBM cells for 24 h merged images of pMLKL (red) and RIP3 (green) colocalize to produce a yellow color, thus suggesting the necrosomes complex formation.

### iPA induces necroptosis in primary glioblastoma cells

**Fig. 6:**
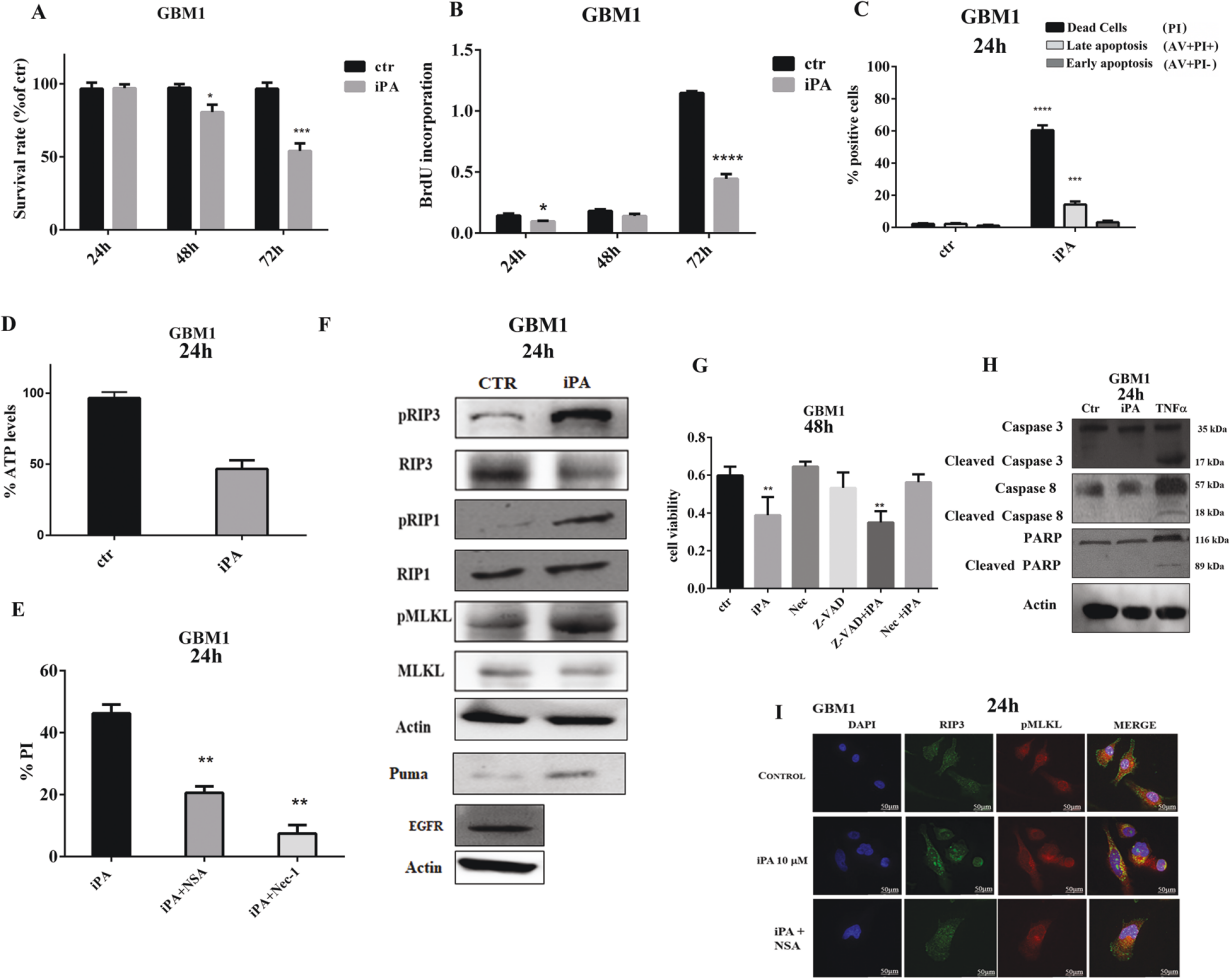
iPA induces necroptosis in GBM primary cell lines. **A** Plot showing the survival rate of GBM1 cells after 24, 48, and 72 h of iPA (10 μM) treatment. **B** Antiproliferative effect of iPA on GBM1 primary cell line as detected by BrdU assay after 24, 48, and 72 h of treatment with iPA (10 μM). **C** GBM1 cell line was treated with 10 µM of iPA for 24 h. Cell death was determined by flow cytometry followed by Annexin V/PI staining. Early Apoptosis Annexin V-positive cells (AV+/PI−); Necrosis: PI-positive and Annexin V-negative cells (AV−/PI+); and Late Apoptosis (AV+/PI+). **D** Plot showing the reduced ATP levels after 24 h of treatment with iPA 10 µM in GBM1 primary cell line. **E** Plot showing the % of PI-positive cells. GBM1 primary cell line after 1 h of pretreatment with Necrosulfonamide (NSA) (5 μM) and Necrostatin-1 (Nec-1) (20 μM) were analysed through PI staining after 24 h of iPA treatment (10 μM). **F** Representative Immunoblotting images of Necroptosis protein levels in GBM1 primary cell line after 24 h of treatment with iPA 10 µM. **G** Plot showing the % of cell viability in GBM1 primary cell line after 48 h of treatment with iPA (10 μM), RIP1 inhibitor Nec-1 (20 μM), Caspase inhibitor z-VAD (10 μM), and two combinations of these drugs: z-VAD (10 μM) + iPA (10 μM) and Nec-1 (20 μM) + iPA (10 μM). Specifically, GBM1 cells were pretreated with z-VAD or Nec-1 for 1 h, followed by treatment with iPA 10 μM for 48 h. **H** Representative Immunoblotting images of Caspase-3, Caspase-8, and PARP1 protein levels in GBM1 primary cell lines treated with iPA 10 µM at 24 h. **I** Immunofluorescence staining of GBM1 cells with RIPK3 (green) and p-MLKL (red) after treatment with iPA 10 µM at 24 h. The nuclei are stained blue with Hoechst. The data are presented as mean ± SD of at least three independent experiments. *p < 0.05, **p < 0.01, ***p < 0.001, ****p < 0.0001 versus control, represented by cells treated with vehicle (DMSO).

To gain insight into the ex vivo effect of iPA, we studied the antitumor potential of iPA in human primary glioblastoma cells. We established one cell model from freshly resected primary tumors from glioma-affected patients. We performed methylome profiling of glioma tissues (GBM WHO IV) and the primary GBM cells derived from the tissues were characterized for the mutational status of IDH1/IDH2 and methylation of MGMT (Table 1) as described in Materials and Methods. To offer a physiological relevance to the experimental features we used primary tumor cells at an early passage (up to the third passage) and low oxygen tension (5% normoxia). We conducted experiments to evaluate the iPA effects on cell viability using a GBM primary cell line (GBM1). We treated GBM1 with 10 µM of iPA for 72 h and assessed cell proliferation by MTT and BrdU assays. As depicted in Fig. 6A and B iPA treatment markedly inhibited tumor cell viability and cell proliferation (50% growth inhibition) at 72 h as compared to the cells treated with the vehicle DMSO. We then studied iPA ability to induce cell death through analysis by Annexin-V and PI staining of GBM1 at 48 h (Fig. 6C). Treatment of GBM1 cells resulted in an increase in the total necrotic and late apoptotic rate suggesting a mechanism of cell death for necroptosis (AV+/PI− (Early Apoptosis):3.9% ± 0.5; AV+/PI+(Late Apoptosis):15.6% ± 1.4; AV−/PI+(Necrosis) iPA:62.6% ± 3.1). No significant effect of the vehicle DMSO on the total necrotic and apoptotic rate was observed. We then tested the ATP levels in GBM1 cells treated with 10 µM iPA for 24 h and we observed a significant decrease in intracellular ATP levels compared to untreated cells (Fig. 6D). Moreover, the PI uptake was inhibited when the GBM1 cells were pretreated with NSA at 5 µM, and with Nec-1 at 20 μM (Fig. 6E). Consistent with data previously reported on GBM stabilized cells, iPA treatment (at 10 µM) induced the activation of necroptosis markers RIP1, RIP3, and MLKL, and the increase of protein levels of HMGB1 and PUMA in GBM1 cells (Fig. 6F). To further confirm that necroptosis was the preferred cell death mechanism observed, GBM1 cells were exposed to apoptosis inhibitor z-VAD-fmk (10 μM) and necroptosis inhibitor Nec-1 (20 μM) for 1 h before iPA (10 μM) treatment. As shown in Fig. 6G, MTT assays results indicated that cell viability indeed decreased significantly after 48 h of iPA treatment, while the survival rate was increased after pretreatment with Nec-1 and was not attenuated by pretreatment with z-VAD-fmk. Following the same experimental course carried out on stabilized cell lines, we excluded the induction of other caspase-dependent cell death mechanisms by observing caspases modulation in GBM1 cells treated with iPA for 24 h compared to cells treated with vehicle alone. As shown in Fig. 6H we did not observe caspase-3, 8, and PARP1 activation in iPA-treated GBM1 cells compared to TNFα-treated cells, suggesting that iPA indeed triggers the necroptotic program in a caspases-independent manner. Finally, as shown in Fig. 6I, immunofluorescence analysis demonstrated again the colocalization (merged, yellow) of pMLKL (red) and RIP3 (green) proteins in iPA-treated GBM1 cells, while the merged images of the same cell line pretreated with NSA did not show any colocalization (yellow color).

**Table 1:** Molecular characteristics of primary cell line.

GBM	Patient		Type	MGMT methylation status		IDH1/IDH2 status		Co-del 1p-19q	Molecular characterization	EGFR amplification	Epigenetic subclass
	Age	Gender		Patient	GBM line	Patient	GBM line				
GBM1	58	F	Primary	Unmethylated	Unmethylated	IDH WT	IDH WT	Absent	GBM IDH WT	Yes	RTKII

Reassuming the epigenetic and genetic profile of GBM1 primary cell line obtained from an affected patient.

## Discussion

Glioblastoma is the most aggressive primary brain cancer, characterized by invasive growth and a poor prognosis. The standard therapeutic approach, known as the Stupp protocol, is surgery followed by radiotherapy and chemotherapy with temozolomide; it improves the 2-years patient survival by only 27% [2]. It is well-established that apoptosis, which is a programmed cell death mechanism, functions as a natural barrier that protects against cancer development. However, the evasion of and resistance to apoptosis are also considered indisputable hallmarks of cancer [17], and resistance to apoptosis is often responsible for both tumorigenesis and drug resistance, resulting in chemotherapy failure. In GBM constitutive EGFR activation due to mutations or gene amplification causes deregulated proliferation, angiogenesis, and inhibition of apoptosis; for this reason, bypassing the apoptotic pathway to induce cancer cell death can be considered a promising approach to overcoming this problem [18–20]. New research is focusing on necroptosis, a programmed cell death, as a potential strategy to eliminate apoptosis-resistant tumor cells. Necroptosis is a regulated necrotic and caspase-independent death program [9]. The role of necroptosis in cancer is complicated. The expression of key regulators of the necroptotic pathway is generally downregulated in cancer cells, suggesting that cancer cells may also evade necroptosis to survive [18]. Although the molecular mechanism has not yet been revealed, current evidence shows that necroptotic cell death requires the activation of RIP1 and RIP3 kinases. When necroptosis is induced, RIP1 is activated and binds to RIP3, determining its oligomerization and autophosphorylation. RIP3 recruits and phosphorylates MLKL, promoting its translocation to the membrane and triggering cell lysis in the necroptotic process. iPA, a cytokinin formed by adenosine harboring an isopentenyl group at N6 position, inhibits the growth of human tumor cell lines in vitro, inducing apoptosis [21, 22]. We previously described that iPA arrests the proliferation of glioblastoma cell lines in vitro and in vivo via downregulation of epidermal growth factor receptor (EGFR) [11]. Based on our present study, we describe for the first time how iPA promotes necroptosis in glioblastoma cell lines: U87MG, the same ones engineered to overexpress EGFR wild-type (wt) or EGFRvIII, and in GBM cells derived from a tumor biopsy of patients affected. In our study, we observed that iPA treatment of GBM cells induces activation of RIP1, RIP3, and MLKL proteins triggering necroptotic cell death. We showed that phosphorylation of the kinases RIPK1, RIPK3, and MLKL occurs at 15 h after iPA incubation. The activation of these kinases is crucial for the necrosome formation and for necroptosis execution [15]. To confirm iPA action, GBM cell death was rescued through specific inhibitors of the proteins involved in the necroapoptotic pathway, NSA (MLKL inhibitor) and Nec-1 (RIP1 inhibitor); indeed the pretreatment with Nec-1 and NSA reduced the rate of necrotic cells detected by flow cytometry with PI staining. On the other hand, the pretreatment with z-VAD-fmk, a general inhibitor of apoptosis, did not elicit a protective effect on cell viability in GBM cells treated with iPA while the pretreatment with Nec-1 inhibits the antiproliferative effect of iPA on GBM cells. We also found that caspase-3, caspase-8, and PARP1 were not activated upon iPA treatment in GBM cells unlike the TNFα treatment inducer of apoptosis, thus clarifying the targeted effect of iPA on the necropoptotic pathway. Necroptosis exhibits other features, including the rupture of the cellular membrane with the release into the extracellular spaces of cytoplasmic molecules such as HMGB1 [23, 24], and the loss of mitochondrial function with subsequently ATP depletion. HMGB1, when released into the extracellular space, functions as a damage-associated molecular pattern (DAMP) acting as a pro-inflammatory stimulus [24]. Importantly, an alteration of its expression has been involved in the response to cancer therapy [11, 12]. Indeed, in our current study, we demonstrated how iPA-treated GBM cells have shown an increase of HMGB1 protein levels as much as depletion of ATP, supporting previous evidence showing that cellular ATP levels are a biochemical hallmark of necroptotic programmed cell death [25, 26].

Chen D et al. show that PUMA, a proapoptotic BH3-only Bcl-2 family member, is induced and plays a role in necroptotic death*;*PUMA is involved in several necroptosis-related diseases such as ischemia-reperfusion [27] and its induction in necroptosis is mediated by MLKL activation [15]. Based on these recent data we analyzed PUMA expression in GBM cells treated with iPA 10 µM. Our data report an increase in the expression of both the mRNA and protein levels of PUMA, proving its contribution to the execution of necroptosis.

**Fig. 7:**
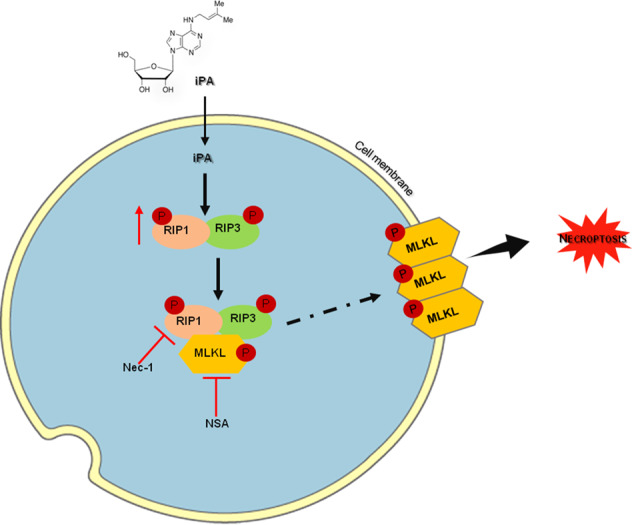
iPA induces necroptosis in GBM cell lines. As showed in our work, iPA can rise the levels of the phosphorylated, and thus activated, forms of RIP1 and RIP3 kinases. The activated proteins can interact with each other forming the necrosome protein complex. The necrosome in turn phosphorylate MLKL, that translocate to the plasma membrane to promote necrosis by disrupting the integrity of cell membrane. Necrostatin-1 (RIP1 inhibitor) and Necrosulfonamide (MLKL inhibitor) were used in this work to prove the induction of necroptosis in GBM treated cell lines by iPA.

All the data obtained so far lead us to conclude that iPA, a natural compound, has an antitumor effect on glioblastoma cells and is able to promote a RIP1 and RIP3-dependent cell death, necroptosis (Fig. 7). In order to develop its clinical use, more studies are required to evaluate the efficacy and safety of iPA alone or in combination with other drugs in the treatment of glioblastoma.

## Materials and methods

### Cell cultures and reagents

The glioblastoma cell lines used for this study are U87MG, purchased from Elab-science (Elabscience, Houston, TX, USA—catalog No. EP-CL-0238); NHA-Astrocytes cells were purchased from Lonza (Rome, Italy—Product Code: CC-2565); U87MG expressing EGFRwt and U87MG expressing EGFRvIII were kindly donated by Professor F.B. Furnari of the Ludwig Institute for Cancer Research and the Moores Cancer Center, University of California, San Diego, La Jolla [28]. Cells were cultured in DMEM (Gibco, Thermo Fisher Scientific, Monza, Italy) supplemented with 10% heat-inactivated fetal bovine serum, 1% L-Glutamine, 1% Sodium Pyruvate, 1% non-essential amino acids (Lonza, Rome, Italy), and 0.1% PlasmocinTM (InvivoGen, San Diego, CA, USA). All cell cultures were maintained at 37 °C in humidified 5% CO_2_ atmosphere. For all the experiments, the solutions were prepared to start from the stock solution. Chemicals used in this article were: N6-isopentenyladenosine (iPA) was purchased from Sigma–Aldrich St. Louis, MO, USA, solubilized in dimethyl sulfoxide (DMSO) and added to cell cultures growth medium at different concentrations. The final concentration of DMSO is 0.1%. The necroptosis inhibitor Necrosulfonamide (NSA) was purchased from Tocris Bioscience (Bristol, UK), solubilized in 2 mL of DMSO, and added to cell cultures at a final concentration of 5 μM for an hour before iPA treatments. Similarly, the necroptosis inhibitor Necrostatin-1 (Nec-1) was purchased from Santa Cruz Biotechnology and added to the cell medium at a final concentration of 20 µM for an hour before iPA treatments. TNF-α was purchased from Sigma–Aldritch (St. Louis, USA) and added to cell culture at a final concentration of 20 ng/mL for an hour before iPA treatment. z-VAD-fmk was purchased from Santa Cruz (Santa Cruz Biotechnology, CA, USA) and added to cell culture at a final concentration of 10 μM for an hour before iPA treatment.

### Preparation of glioblastoma primary cell lines

For primary cell lines, the patients underwent tumor resection at the Neurosurgery Service of “Antonio Cardarelli” Medical Hospital (Naples, Italy). The tumor biopsies were immediately processed to obtain primary tumor cell lines. A second sample derived from each patient was taken for clinical diagnosis, according to the International Classification of CNS tumors drafted under the auspices of the World Health Organization (WHO). All tissue samples were collected in accordance with the ethical standards of the Institutional Committee (DEL. N°897 August 13, 2020). Informed consent in written form was obtained from all subjects involved in the study. As for the preparation of adherent primary cultures (designated as GBMn), the tumors were cut into small pieces and minced. The minced samples were prepared using the gentleMACS™ Dissociator in combination with the Tumor Dissociation Kit, human (Miltenyi Biotec, Cologne, Germany. Cod# 130-095-929). After the preparation of the enzymes mix, the tumor samples were transferred into the gentleMACS C Tube. Once the C Tube was attached to the sleeve of gentleMACS Dissociator, different programs were run according to the protocol. The obtained cell suspension was then applied to a MACS SmartStrainer, mesh size 70 μm, placed on a 50 mL tube. The cell strainer was washed with 20 mL of RPMI 1640 and the cell suspension was centrifuged at 300 × *g* for 5 min. The supernatant was completely aspirated, the cells resuspended, and then cultured in DMEM/Ham’s F-12 supplemented 15% heat-inactivated Fetal Bovine Serum, 2% L-Glutamine, 1% Sodium Pyruvate, 1% non-essential amino acids (Lonza, Rome, Italy), and 1.5% D-Glucose.

### MTT viability and BrdU assays

Cells were plated at a density of 9 × 10^3^ cells/cm^2^ in 96-wells plate After treatment with iPA dissolved in culture medium (DMEM), the cells were washed once with PBS and incubated with a new medium in which 0.5 mg/ml of MTT (Sigma–Aldrich, St. Louis, USA) was dissolved for sufficient time for the formation of purple crystals. Subsequently, the medium was removed and acid-isopropanol (10% HCl 1 N in isopropanol) was added to each plate to solubilize the crystals. After 20 min at room temperature in agitation, the samples were recovered and cell mortality was assessed with a Synergy HT Microplate Reader (BioTek Instruments Inc., Winooski, VT, USA) using a wavelength of 570 nm. For BrdU assay, the experiment was performed using the 5-bromo-2’-deoxyuridine ELISA kit (Roche, Basel, Switzerland) according to the following instructions. The cells were seeded in a 96-well plate and incubated with iPA at the indicated concentrations for 24 and 48 h of treatment. Subsequently, the cells were incubated for about two hours with 10 µL of BrdU/well, added to the medium at a concentration of 100 µM (BrdU Labeling Solution diluted 1: 100 in sterile medium). After removing the culture medium, 100 µL of FixDenant included in the Kit was added per well to fix the cells. After an incubation period of 30 min, the FixDenant solution was removed, the cells were incubated for ~90 min with 100 µL / well of anti-BrdU-POD diluted according to instructions. After the cells were washed with PBS 1× to remove the unbound antibody, 100 µl of Substrate Solution was added. The absorbance values were measured with Synergy HT Microplate Reader (BioTek Instruments Inc., Winooski, VT, USA) at a wavelength of 450 nm.

### ATP assay

The CellTiter-Glo® Luminescent Cell Viability Assay kit (Promega Italia s.r.l, Milano, Italy) was used to evaluate the ATP levels. For the execution of the assay, 2.5 × 10^4^ cells were seeded in 96-well plates. After equilibrating the plate and the cells at room temperature for about 30 min, 100 µL of CellTiter-Glo® Reagent were added per well. After leaving the plate in agitation for ~2 min to allow cell lysis, the plate was left at room temperature for 10 min to stabilize the luminescent signal, which was subsequently evaluated by BioTek’s Synergy™ HT Luminometer (BioTek Instruments Inc., Winooski, VT, USA).

### RNA isolation and quantitative RT-PCR

For the RNA extraction, cells were seeded on p60 dishes at a density of 2 × 10^6^ cells/cm^2^ in supplemented DMEM medium and incubated with iPA. Total RNA was then isolated using EuroGold Trifast reagent (EuroClone, Pavia, Italy) according to the manufacturer’s instructions. The obtained RNA samples, resuspended in 20 μL of sterile water, were measured using a NanoDrop spectrophotometer (Thermo Fisher Scientific, Monza, Italy) at a wavelength of 260 nm. The RNA was then reverse transcribed into cDNA using superScript II reverse transcriptase (Invitrogen, Carlsbad, CA, USA) starting from 1 µg of highly purified RNA. Quantitative RT-PCR was performed using gene-specific primers and a SYBR Green I (Promega Italia s.r.l, Milano, Italy) fluorescent dye. The sequences of gene-specific primers are shown in Table 2.

**Table 2:** List of primers used.

Gene	Forward oligo	Reverse oligo
*RIP1*	5’- GGC ACC GCT AAG AAG AAT GG- 3’	5’- ATC GCC CAG AGT ACT ACA GC- 3’
*RIP3*	5’- ATA CAA CTG CTC TGG GGT GC -3’	5’- TCT TGC GAA CCT ACT GGT GG -3’
*MLKL*	5’- AGG ACC AAG GAA AGA GGA GC- 3’	5’-TGT CCT TTG CTG TTA GAC -3’
*HMGB1*	5’- CAA GTA AAT GGA AGT GGG AGG C-3’	5’- AAC CCC ACA GCA CTG TAA CT-3’
*PUMA*	5’- AAT GAG CCA AAC GTG ACC AC- 3’	5’- GCA GAG CAC AGG ATT CAC AG -3’
*β*_*2-MICROGLOBULIN*_	5’-CCT GAA TTG CTA TGT GTC TGG G-3’	5’-ACA CGG CAG GCA TAC TCA TC -3’

### Flow cytometry

The cells were plated at a density of 5 × 10^5^ cells/cm^2^ in p60 dishes in supplemented DMEM for about 24 h. Subsequently, they were treated at the indicated concentrations and times. Assessment of apoptosis was conducted by human anti-annexin V staining and propidium iodide (PI) (Dojindo Molecular Technologies, MD, USA). Cells were harvested with trypsin and washed in PBS, resuspended in annexin V binding buffer (10 mM Hepes / NaOH, Ph 7; 140 mM NaCl; 2.5 mM CaCl2), stained with annexin V-FITC for 20 min at room temperature and then stained with PI at room temperature for a further 15 min in the dark. Cells were acquired by flow cytometer within 1 h of staining. At least 10,000 events were collected and the data were analyzed by BD Accuri C6 software. For cell cycle analysis, after iPA treatment cells were harvested, washed twice with ice-cold PBS 1×, fixed in cold 70% ethanol, and kept at −20 °C overnight. Cells were labeled with PI (50 µg/ml) in the presence of RNase at 37 °C for 30 min at room temperature in the dark. The stained cells were subjected to flow cytometry analysis and the cell cycle distribution was analyzed by BD Accuri C6 software.

### Western blot analysis

Cells were grown in p60 plates at a density of 2 × 10^6^ cells/cm^2^, treated with iPA diluted in a growth medium, and subsequently recovered. To obtain total protein lysates, treated cells were lysed in cold RIPA lysis buffer (50 mM Tris-HCl, 150 mM NaCl, 0.5% Triton X-100, 0.5% deoxycholic acid, 10 mg / mL leupeptin, 2 mM phenylmethylsulfonyl fluoride, and 10 mg/mL aprotinin containing protease and phosphatase inhibitors) (Sigma–Aldrich, St. Louis, USA). Samples were quantified using Protein Analysis Dye Reagent Concentrate (BioRad, Hercules, CA, USA). Proteins were loaded into wells, separated on SDS-PAGE gels at different percentages, transferred to Nitrocellulose membranes using a Trans-Blot® Turbo™ Transfer System (BioRad, Hercules, CA, USA), saturated, and blocked with 5% fat-free milk in Tris saline buffer containing 0.1% Tween-20 (TBST) for 1 h and incubated overnight at 4 °C with specific antibodies. The signals were detected using the ChemiDoc MP image sensor (BioRad, Hercules, CA, USA) after the membranes were soaked in enhanced ECL reagents (Amersham, GE Healthcare, UK). Some membrane signals were captured by exposure to X-ray film (Santa Cruz Biotechnology, CA, USA). Total extracts were normalized using an anti-β-actin antibody. The secondary antibodies used in these experiments were either goat anti-rabbit or goat anti-mouse IgG conjugated with horseradish peroxidase (BioRad, Hercules, CA, USA). The following antibodies were used for the western blot analysis: mouse monoclonal anti-human β-actin, goat monoclonal anti-human Caspase-8, mouse monoclonal anti-human MLKL, mouse monoclonal anti-human RIP1, and mouse monoclonal anti-human RIP3 were purchased from Santa Cruz Biotechnology (CA, USA); rabbit monoclonal anti-human pMLKL (phospho S358), rabbit monoclonal anti-human HMGB1, rabbit monoclonal anti-human pRIP3 (phospho Ser227) and rabbit monoclonal anti-human PUMA purchased from Abcam (Cambridge, UK); rabbit monoclonal anti-human EGF Receptor, rabbit monoclonal anti-human pRIP1 (phosphor Ser166), rabbit monoclonal anti-human Caspase-3 and rabbit monoclonal anti-human PARP from Cell Signaling Technology (Danvers, United States). The company and concentrations of all antibodies used are presented in Table 3.

**Table 3:** List of antibodies used.

Primary antibodies	Company	Dilution
*β-Actin*	Santa Cruz Biotechnology (sc-47778); monoclonal anti-mouse antibody	1:1000
*Phospho-MLKL*	Abcam (ab187091); monoclonal anti-rabbit antibody; Ser358 phosphorylated	1:1000
*MLKL*	Santa Cruz Biotechnology (sc-293201); monoclonal anti-mouse antibody	1:1000
*Phospho-RIP1*	Cell Signaling (#65746); monoclonal anti-rabbit antibody; Ser166 phosphorylated	1:1000
*RIP1*	Santa Cruz Biotechnology (sc-133102); monoclonal anti-mouse antibody	1:1000
*Phospho-RIP3*	Abcam (ab 209384); monoclonal anti-rabbit antibody; Ser227 phosphorylated	1:1000
*RIP3*	Santa Cruz Biotechnology (sc-374639); monoclonal anti-mouse antibody	1:1000
*HMGB1*	Abcam (ab77302); monoclonal anti-rabbit antibody	1:1000
*PUMA*	Santa Cruz Biotechnology (sc-377015); monoclonal anti-mouse antibody	1:1000
*EGFR*	Cell Signaling (#4405 S); monoclonal anti-rabbit antibody	1:1000
Caspase-3	Cell Signaling (#9662); monoclonal anti-rabbit antibody	1:1000
Caspase-8	Santa Cruz Biotechnology (sc-6136); polyclonal anti-goat antibody	1:1000
PARP	Cell Signaling (#9532); monoclonal anti-rabbit antibody	1:1000

Secondary antibodies	Company	Dilution
Goat Anti-Rabbit HRP	Bio Rad	1:5000
Goat Anti-Mouse HRP	Bio Rad	1:5000

### Immunofluorescence

Cells were seeded on coverslips in 24-well culture plates at a density of 1 × 10^5^ cells/cm^2^ and treated with iPA for 24 h at 37 °C. After, were fixed with 3.7% paraformaldehyde, permeabilized with 0.2% Triton X-100 and blocked using PBS-BSA 0.4%. Then, cells were incubated with primary antibody at 4 °C overnight. Following washes with PBS 1× for three times, cells were incubated with a labeled secondary antibody at room temperature for 1 h. Nuclei were then stained with DAPI (Hoechst, Life Technologies Corporation). Finally, cells were washed with PBS 1× for three times and mounted on the slide using Dako Fluorescent Mounting Medium. The images were acquired using a Leica Thunder Imaging System (Leica Microsystems Srl, Buccinasco, Italy) equipped with Leica DFC9000GTC camera and a planapo ×100 oil immersion (NA 1.4) objective lens. Images were acquired by using the Small Volume Computational Clearing (SVCC) mode. For fluorescence microscopy, the following were used as primary antibody: mouse monoclonal anti-human RIP3 (Santa Cruz Biotechnology, CA, USA; dilution 1:200), rabbit monoclonal anti-human pMLKL (Abcam, Cambridge, UK; dilution 1:200); secondary antibody: Alexa Fluor® 488 goat polyclonal anti-rabbit IgG (Jackson ImmunoResearch Europe Ltd., Cambridge, UK; dilution 1:200), DyLight® 594 goat polyclonal anti-mouse IgG (Abcam, Cambridge, UK; dilution 1:200).

### Primary glioblastoma characterization

We used Illumina EPIC ARRAY 850 Beads-Chip (850 K) to evaluate the DNA methylation status of 850,000 CpG sites, for each tumor sample, according to the manufacturer’s instructions. The epigenomic profile was compared to a reference cohort previously analyzed at the German Cancer Research Center using a specific algorithm and customized bioinformatics packages as described previously [29]. In addition, the array data were used to calculate a copy number variation profile, as previously described [30]. In this study, samples Copy number profiles were used to verify the presence/absence of EGFR gene. “Gain” or “amplification” was determined by log2 > 0.3 [14].

### IDH1 and IDH2 mutation status

Tumor DNA extracted from FFPE tumor tissue (FFPE Tissue Kit, Qiagen S.r.l., Milano, Italy) was amplified using specific primers for exon 4 of IDH1 and IDH2 genes. (IDH1: forward primer 5’-TGTAAAACGACGGCCAGTGGATGCTGCAGAAGCTATAA-3’; reverse primer 5’-CAGGAAACAGCTATGACCTTCATACCTTGCTTAATGGG-3’. IDH2: forward primer 5’-TGTAAAACGACGGCCAGTAATTTTAGGACCCCCGTCTG-3’; reverse primer 5’-CAGGAAACAGCTATGACCGGGGTGAAGACCATTTTGAA-3’). Codon 100 and 132 of IDH1 and codon 140 and 172 of IDH2 were then analyzed by Sanger sequencing method [14].

### MGMT methylation assessment

MGMT promoter methylation was determined by Methylation-specific PCR (MSP). DNA extracted from Tumor samples was converted by sodium bisulfite with EZ DNA Methylation Gold Kit (Zymo Research) according to the manufacturer’s protocol. Methylation-Specific PCR was performed using a Nested PCR. The first PCR was performed using specific primers 5’-GGATATGTTGGGATATAGTT-3’, reverse: 5’-CCATCCACAATCACTACAA). In the second PCR step we used different primers for methylated and non-methylated DNA samples (Methylated MGMT: forward primer 5’-TTTCGACGTTCGTAGGTTTTCGC-3’; reverse primer 5’- GCACTCTTCCGAAAACGAAACG-3’. Unmethylated MGMT: forward primer 5’-TTTGTGTTTTGATGTTTGTAGGTTTTTGT-3’; reverse primer 5’-AACTCCACACTCTT CCAAAAACAAAACA-3’) as previously described [31]. As a positive control, we used a commercial methylated DNA for methylated MGMT alleles, and as a negative control, we used a non-methylated commercial control (EpiTEC controls from Qiagen S.r.l., Milano, Italy). Controls without DNA were used for each set of MSP assays. Then, each MSP product was loaded directly onto 3% percent agarose gel, stained with ethidium bromide (Sigma–Aldrich, St. Louis, USA), and examined under ultraviolet illumination using a ChemiDoc MP image sensor (BioRad, Hercules, CA, USA) [14].

### Statistical analysis

Statistical analysis was performed in all the experiments shown by using the GraphPad prism 7.0 software for Windows (GraphPad Software, San Diego, CA, USA). For each type of assay or phenotypic analysis, data obtained from multiple experiments are calculated as mean SD and analyzed for statistical significance using the two-tailed Student’s *t*-test, for independent groups, or one ANOVA and two ANOVA followed by Bonferroni correction for multiple comparisons. *p*-values *p < 0.05, **p < 0.01, and ***p < 0.001 versus control, represented by cells treated with vehicle (DMSO) were considered significant. All experiments were performed in triplicate and repeated three and five times.

## Supplementary information

Raw data

Figure S1

## Data Availability

The data presented in this study are available on request from the corresponding author.
