# Supplementary material for: N6-isopentenyladenosine induces cell death through necroptosis in human glioblastoma cells
Source: Cell Death Discov. 2022 Apr 7;8:173. doi: 10.1038/s41420-022-00974-x (PMC8991250; doi:10.1038/s41420-022-00974-x)
Supplement: Supplementary file 1 — Raw data [file 41420_2022_974_MOESM1_ESM.pdf]

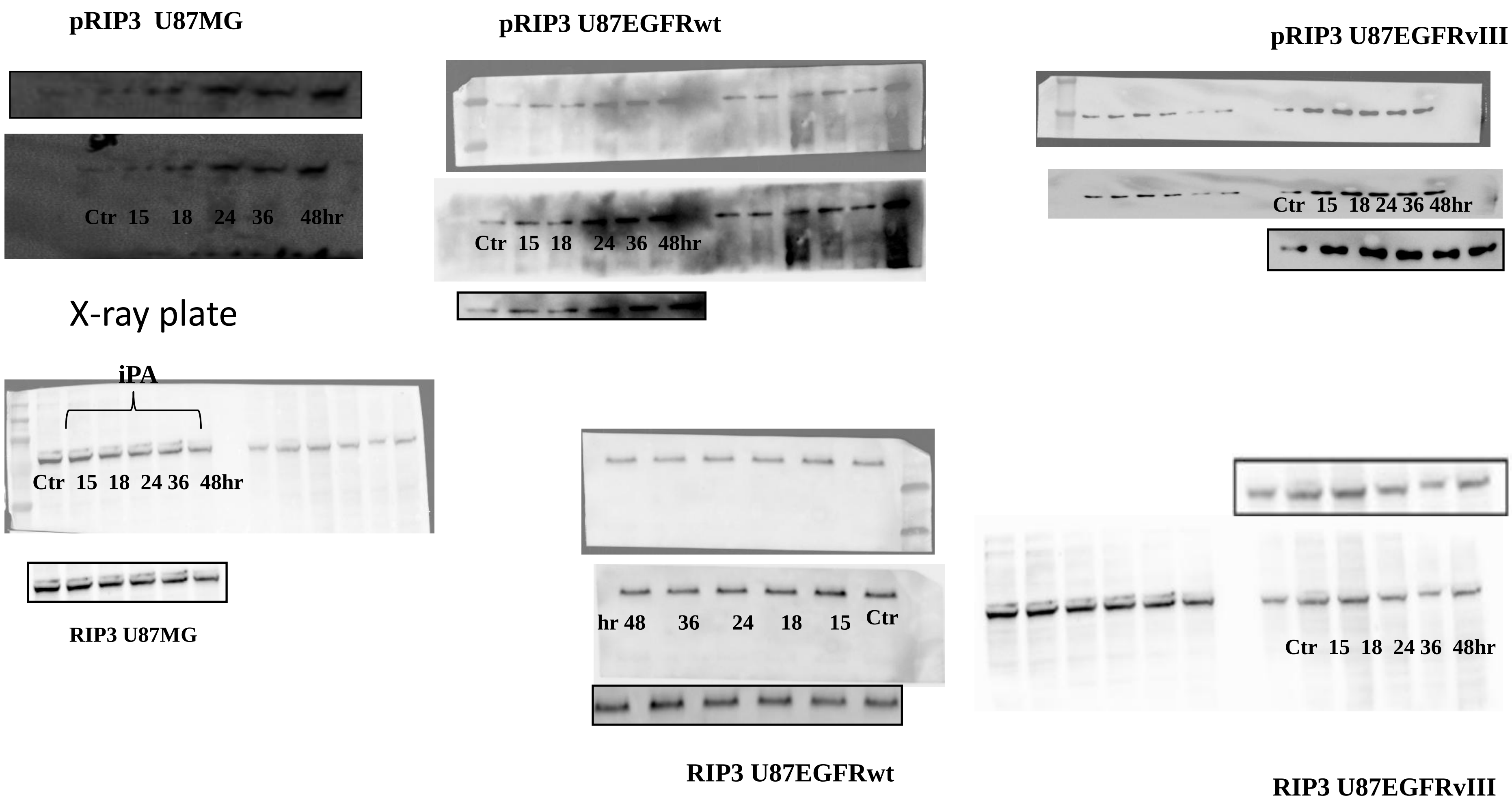
pRIP3 U87MG
Ctr 15 18 24 36 48hr
X-ray plate
pRIP3 U87EGFRwt
Ctr 15 18 24 36 48hr
pRIP3 U87EGFRvIII
Ctr 15 18 24 36 48hr
iPA
Ctr 15 18 24 36 48hr
RIP3 U87MG
hr 48 36 24 18 15 Ctr
RIP3 U87EGFRwt
Ctr 15 18 24 36 48hr
RIP3 U87EGFRvIII

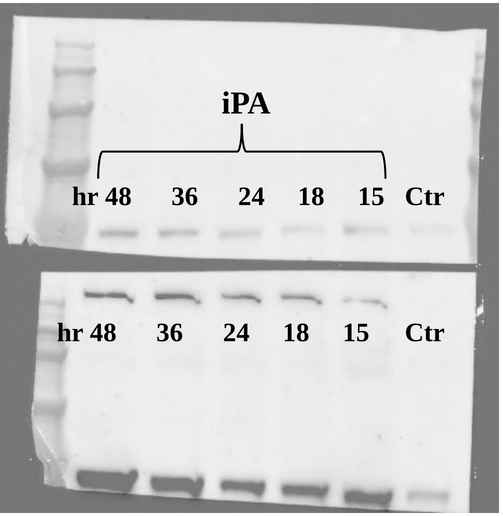

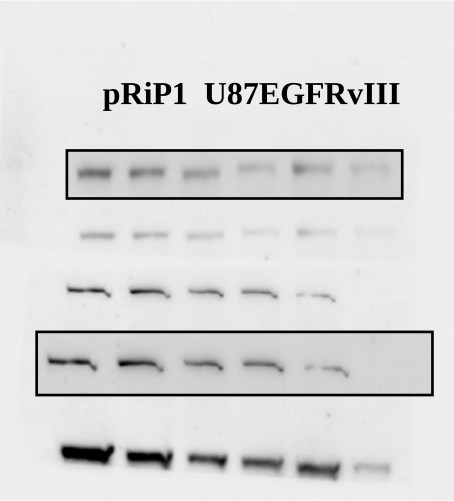

pRiP1 U87MG

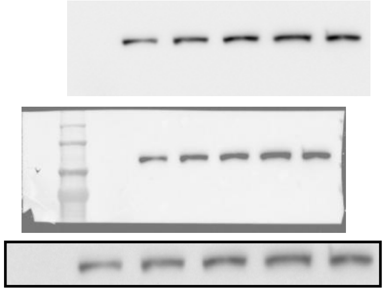

Ctr 15 18 24 36 48hr

pRiP1 EGFRwt

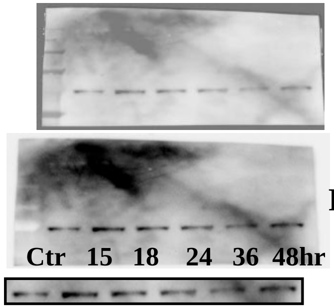

Rip1 U87EGFRvIII

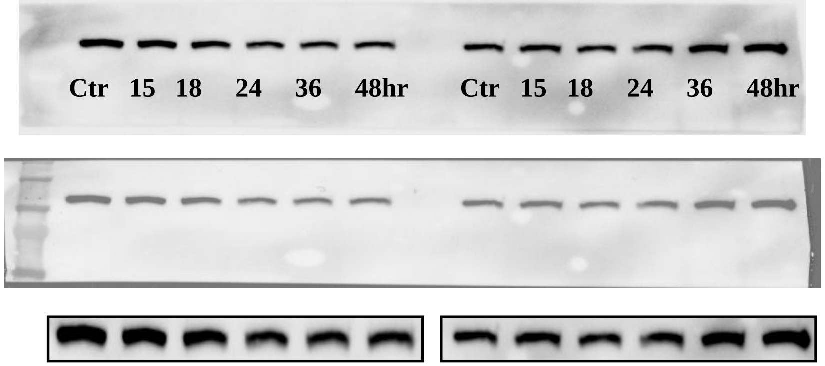

Rip1 U87MG

Rip1 U87EGFRwt

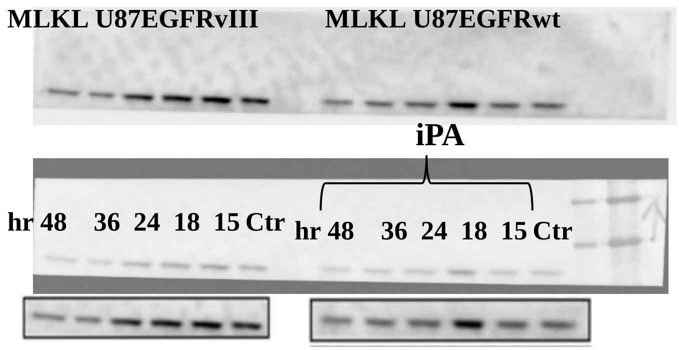

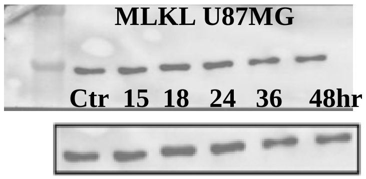

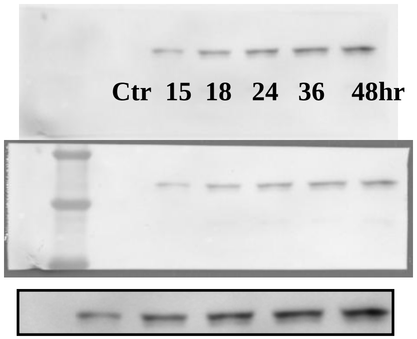

pMLKL U87EGFRwt

CTR

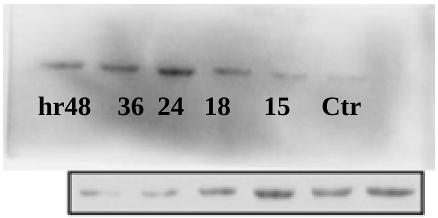

pMLKL U87EGFRwt

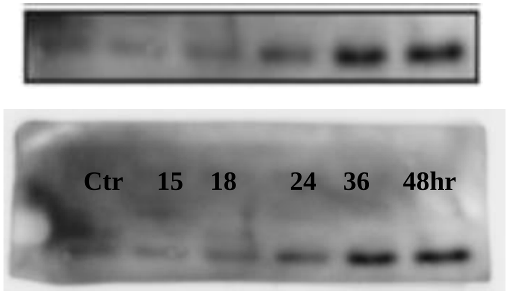

pMLKL U87EGFR vIII

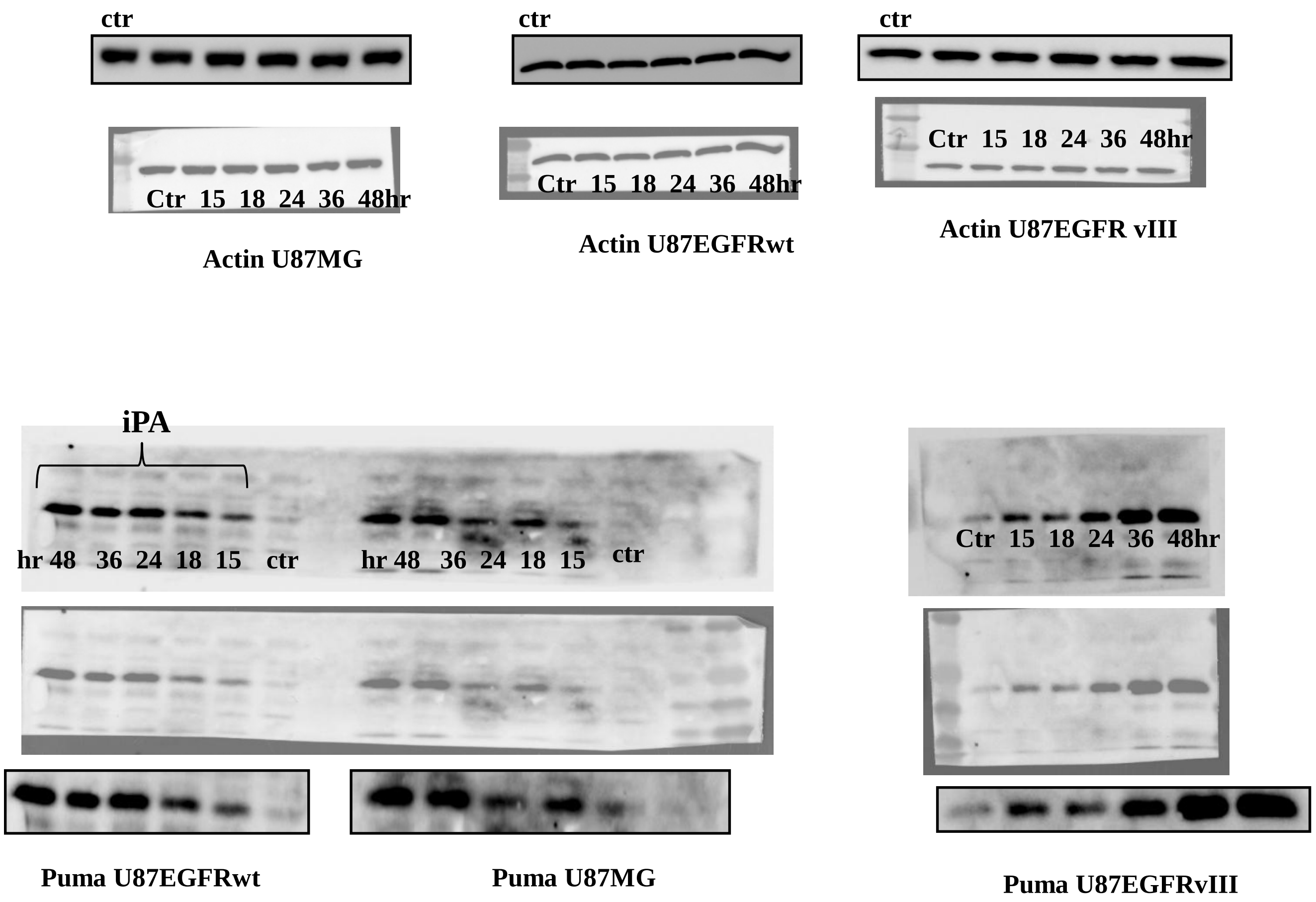
ctr
ctr
ctr
Ctr 15 18 24 36 48hr
Ctr 15 18 24 36 48hr
Ctr 15 18 24 36 48hr
Actin U87MG
Actin U87EGFRwt
Actin U87EGFR vIII
iPA
hr 48 36 24 18 15 ctr
hr 48 36 24 18 15 ctr
Ctr 15 18 24 36 48hr
Puma U87EGFRwt
Puma U87MG
Puma U87EGFRvIII

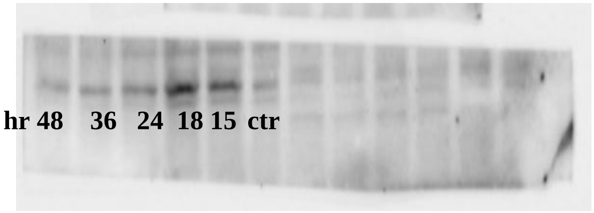

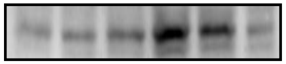

HMGB1 U87EGFR vIII

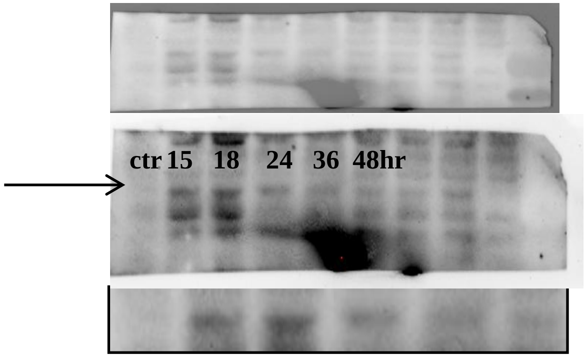

HMGB1 U87EGRFRwt

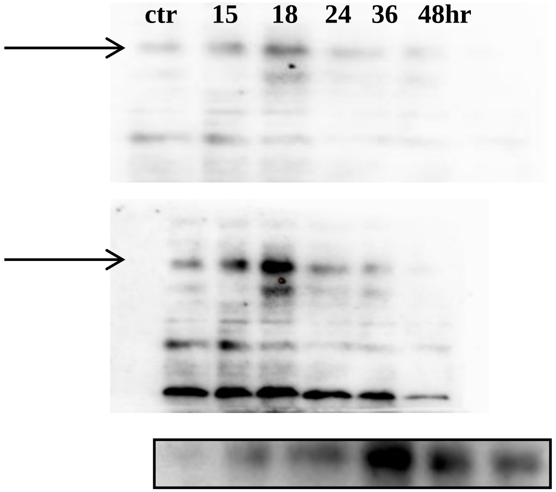

HMGB1 U87MG

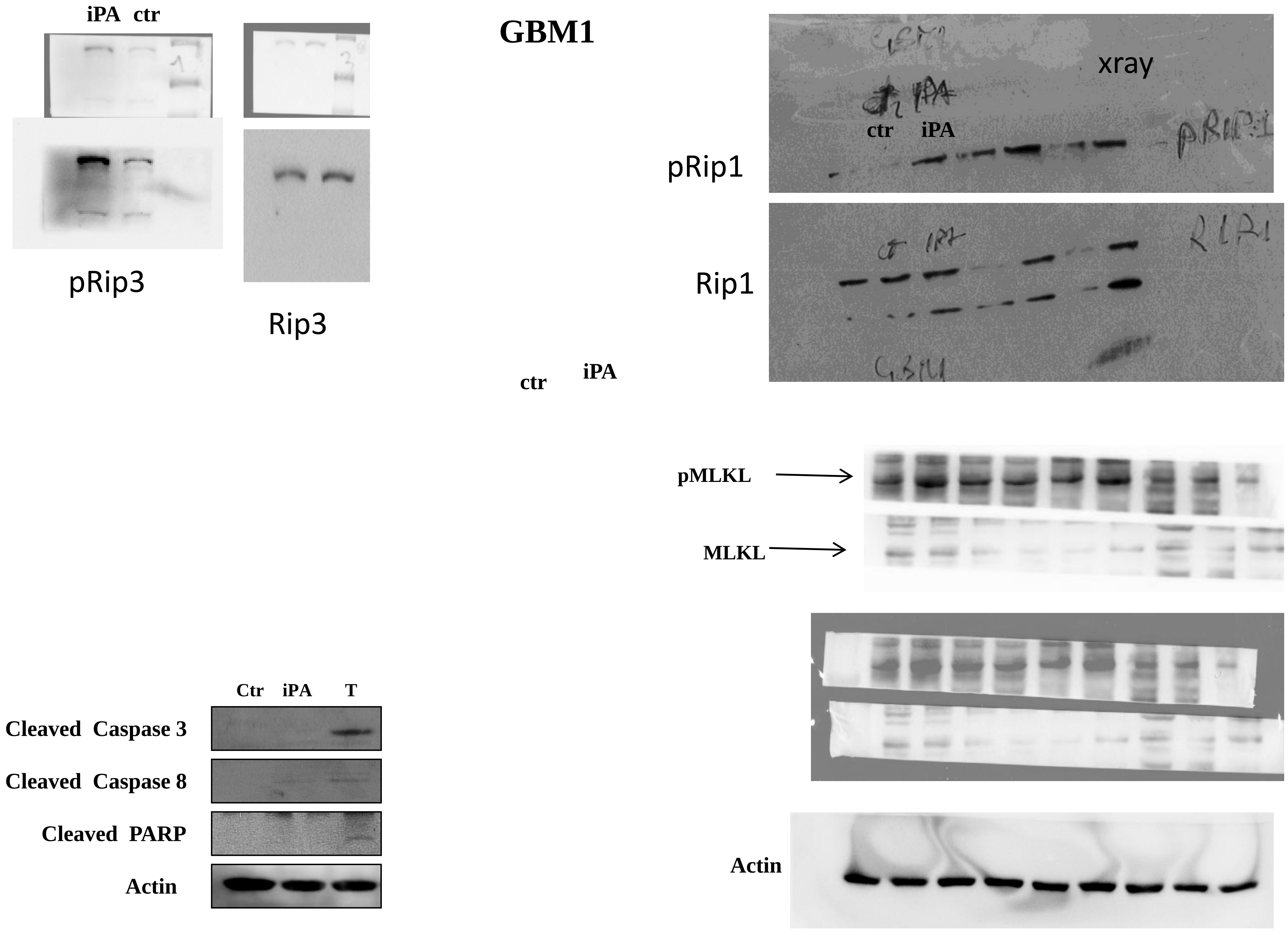
GBM1
iPA ctr
pRip3
Rip3
xray
ctr iPA
pRip1
Rip1
ctr iPA
pMLKL
MLKL
Actin
Ctr iPA T
Cleaved Caspase 3
Cleaved Caspase 8
Cleaved PARP
Actin

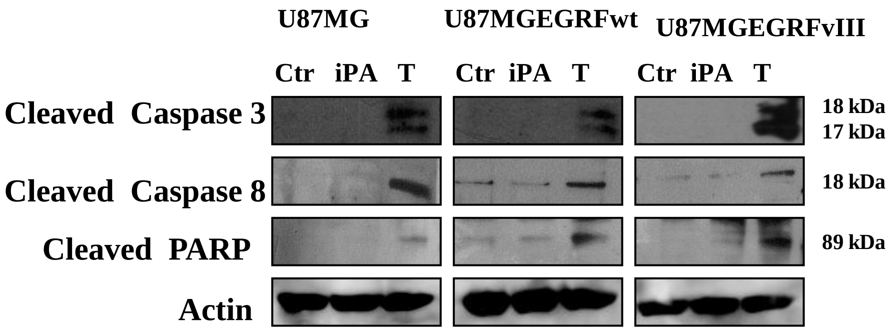
U87MG
U87MGEGRFwt
U87MGEGRFvIII
Ctr iPA T
Ctr iPA T
Ctr iPA T
Cleaved Caspase 3
Cleaved Caspase 8
Cleaved PARP
Actin
18 kDa
17 kDa
18 kDa
89 kDa

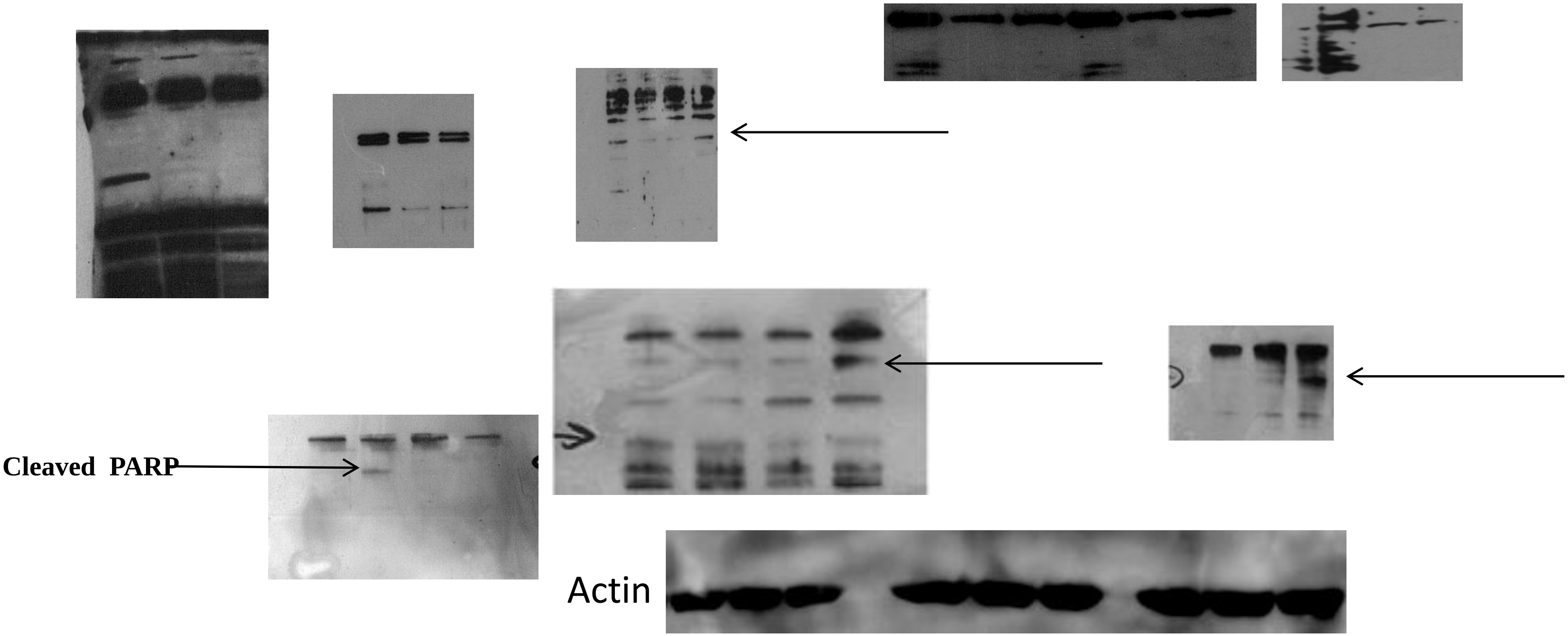
Cleaved PARP
Actin

GBM1

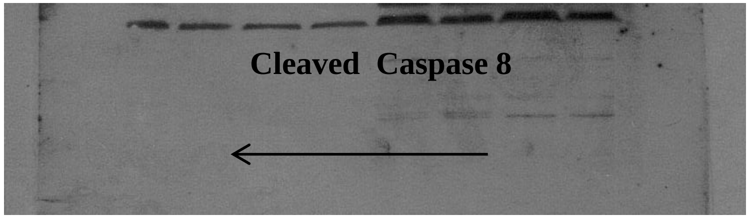

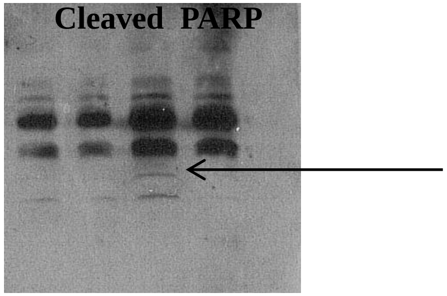

Caspase 3

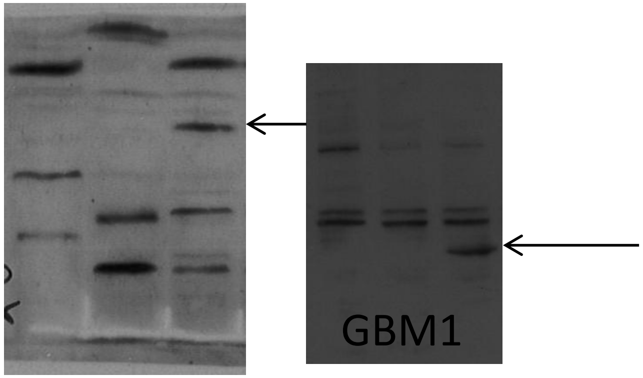

GBM1

GBM1

Actin

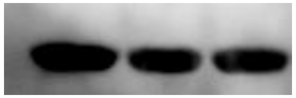

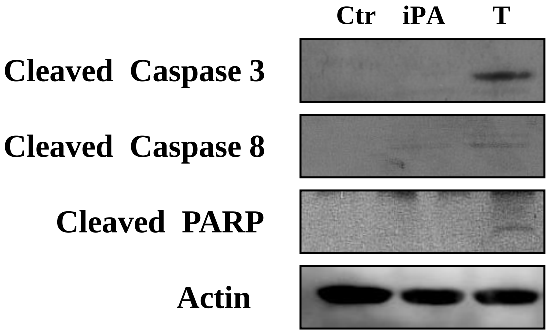

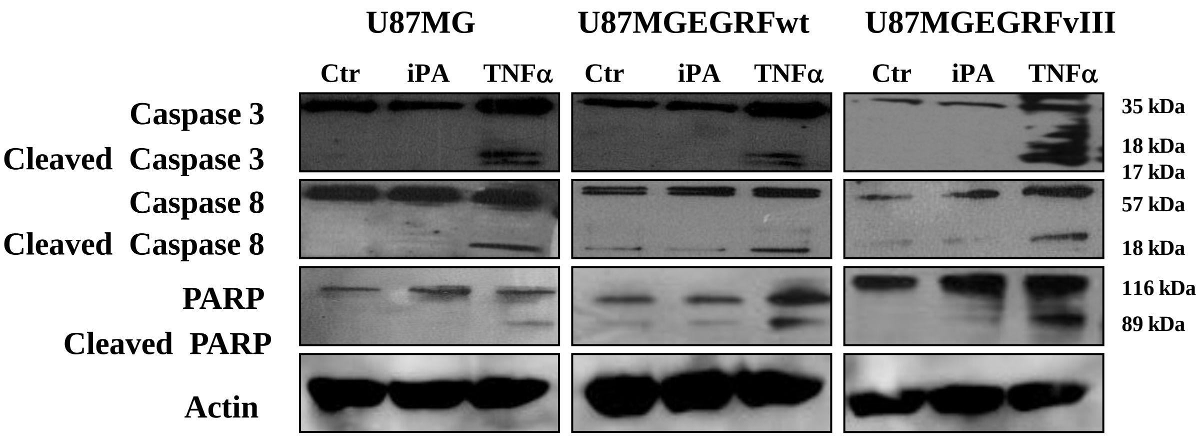
U87MG
U87MGEGRFwt
U87MGEGRFvIII
Ctr
iPA
TNFα
Caspase 3
Cleaved Caspase 3
Caspase 8
Cleaved Caspase 8
PARP
Cleaved PARP
Actin
35 kDa
18 kDa
17 kDa
57 kDa
18 kDa
116 kDa
89 kDa

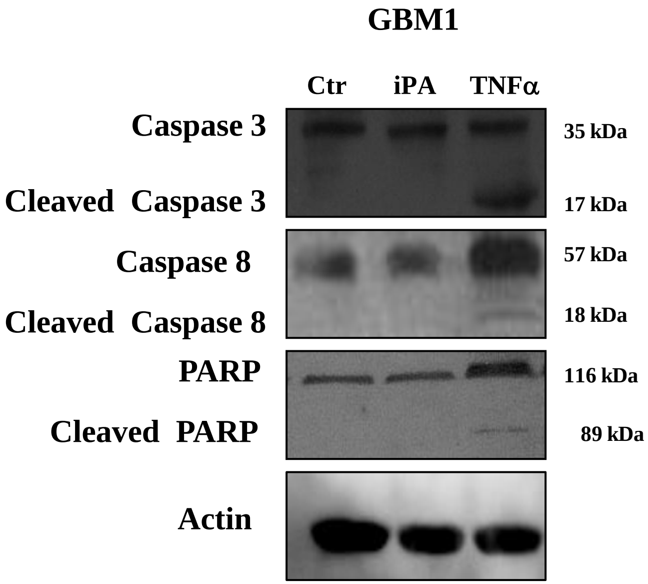
GBM1
Ctr
iPA
TNFα
Caspase 3
Cleaved Caspase 3
Caspase 8
Cleaved Caspase 8
PARP
Cleaved PARP
Actin
35 kDa
17 kDa
57 kDa
18 kDa
116 kDa
89 kDa
