# Supplementary figures and images for: N6-isopentenyladenosine induces cell death through necroptosis in human glioblastoma cells
Source: Cell Death Discov. 2022 Apr 7;8:173. doi: 10.1038/s41420-022-00974-x (PMC8991250; doi:10.1038/s41420-022-00974-x)

|  |  |  |
| --- | --- | --- |

**
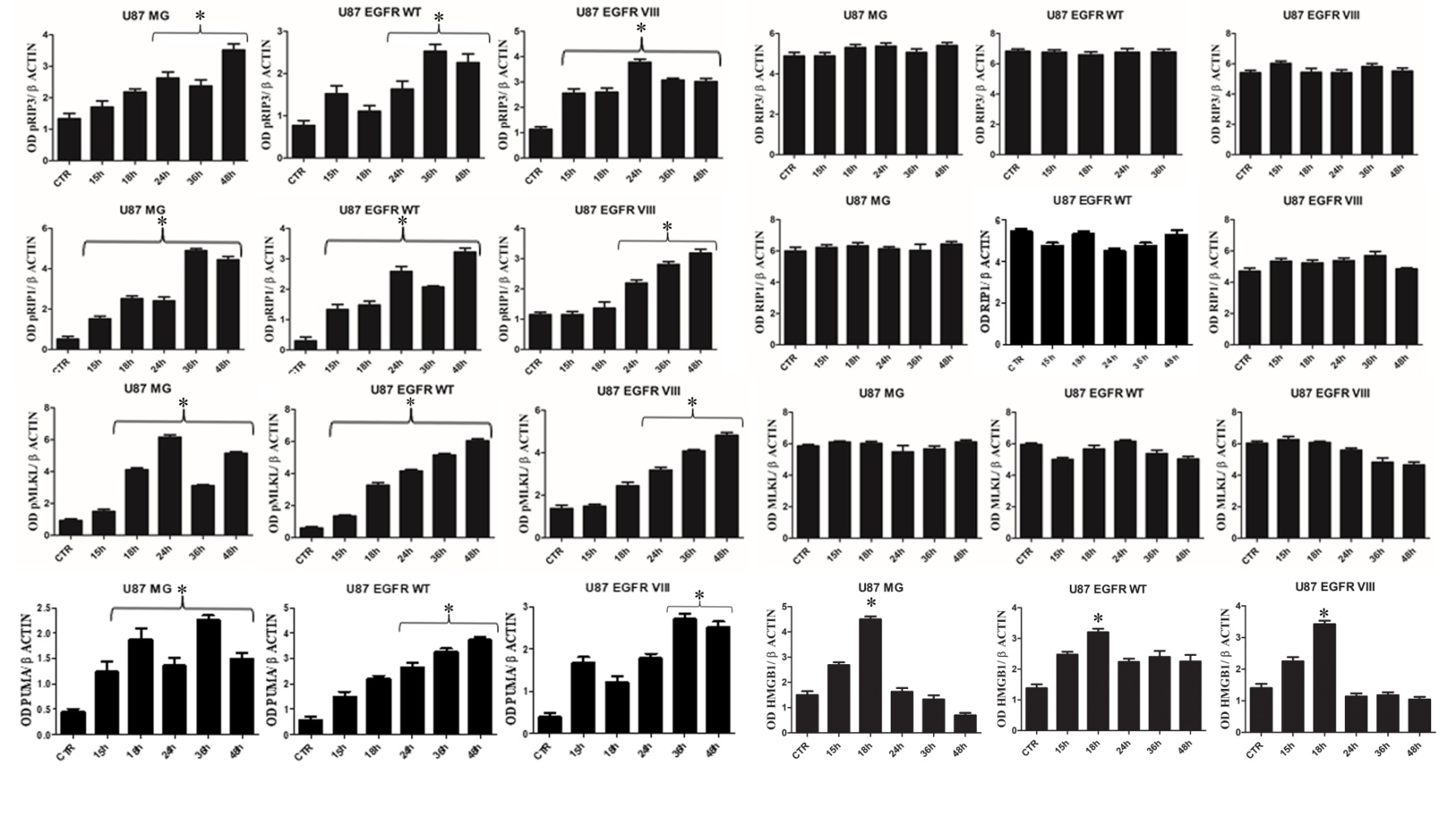
**

**Figure S1** :The densitometry analysis of each blot of Figure 4 A

Supplement: Supplementary file 2 — Figure S1 [file 41420_2022_974_MOESM2_ESM.docx]
